# Detection of deviance in Japanese kanji compound words

**DOI:** 10.3389/fnhum.2022.913945

**Published:** 2022-08-15

**Authors:** Yuka Egashira, Yoshimi Kaga, Atsuko Gunji, Yosuke Kita, Motohiro Kimura, Naruhito Hironaga, Hiroshige Takeichi, Sayuri Hayashi, Yuu Kaneko, Hidetoshi Takahashi, Takashi Hanakawa, Takashi Okada, Masumi Inagaki

**Affiliations:** ^1^Department of Developmental Disorders, National Institute of Mental Health, National Center of Neurology and Psychiatry (NCNP), Kodaira, Japan; ^2^Department of Pediatrics, Faculty of Medicine, University of Yamanashi, Chuo, Japan; ^3^College of Education, Yokohama National University, Yokohama, Japan; ^4^Integrative Brain Imaging Center, National Center of Neurology and Psychiatry (NCNP), Kodaira, Japan; ^5^Cognitive Brain Research Unit (CBRU), Faculty of Medicine, University of Helsinki, Helsinki, Finland; ^6^Department of Psychology, Faculty of Letters, Keio University, Minato-ku, Japan; ^7^Department of Information Technology and Human Factors, National Institute of Advanced Industrial Science and Technology (AIST), Tsukuba, Japan; ^8^Brain Center, Faculty of Medicine, Kyushu University, Fukuoka, Japan; ^9^Open Systems Information Science Team, Advanced Data Science Project, RIKEN Information R&D and Strategy Headquarters (R-IH), RIKEN, Yokohama, Japan; ^10^Department of Neurosurgery, National Center Hospital, National Center of Neurology and Psychiatry (NCNP), Kodaira, Japan; ^11^Department of Child and Adolescent Psychiatry, Kochi Medical School, Kochi University, Nankoku-shi, Japan; ^12^Integrated Neuroanatomy and Neuroimaging, Kyoto University Graduate School of Medicine, Kyoto, Japan; ^13^Department of Pediatrics, Tottori Prefectural Rehabilitation Center, Tottori, Japan

**Keywords:** visual word processing, kanji compounds, MEG, reading ability, parafoveal vision, lexical processing, automatic processing

## Abstract

Reading fluency is based on the automatic visual recognition of words. As a manifestation of the automatic processing of words, an automatic deviance detection of visual word stimuli can be observed in the early stages of visual recognition. To clarify whether this phenomenon occurs with Japanese kanji compounds—since their lexicality is related to semantic association—we investigated the brain response by utilizing three types of deviants: differences in font type, lexically correct or incorrect Japanese kanji compound words and pseudo-kanji characters modified from correct and incorrect compounds. We employed magnetoencephalography (MEG) to evaluate the spatiotemporal profiles of the related brain regions. The study included 22 adult native Japanese speakers (16 females). The abovementioned three kinds of stimuli containing 20% deviants were presented during the MEG measurement. Activity in the occipital pole region of the brain was observed upon the detection of font-type deviance within 250 ms of stimulus onset. Although no significant activity upon detecting lexically correct/incorrect kanji compounds or pseudo-kanji character deviations was observed, the activity in the posterior transverse region of the collateral sulcus (pCoS)—which is a fusiform neighboring area—was larger when detecting lexically correct kanji compounds than when detecting pseudo-kanji characters. Taken together, these results support the notion that the automatic detection of deviance in kanji compounds may be limited to a low-level feature, such as the stimulus stroke thickness.

## Introduction

Mechanisms underlying effortless reading, such as the processes of immediate and automatic word recognition, remain unclear (Schotter et al., 2011; Dawson et al., 2018). Elucidating such mechanisms is clinically important and will contribute to establishing non-pharmacological therapeutic measures. Recent studies have proposed an empirical theory regarding the learning deficits in dyslexia and the benefits of brain stimulation in reducing this reading difficulty (Turker and Hartwigsen, 2021, 2022). Previous event-related potential (ERP) studies have indicated that the lexical information of words in a reader’s native language is processed early and automatically within 400 ms of stimulus onset (Shtyrov et al., 2013; Wei et al., 2018). Enhanced ERPs were reported upon the pre-attentive presentation of deviant words in Russian (Shtyrov et al., 2013); equivalent findings were reported for Chinese character deviance presentation to Chinese speakers, but not to non-Chinese speakers (Wei et al., 2018). However, the specific aspects of lexicality, or dimensions of the contrasts between visually presented words and non-words, such as orthography or semantics (Coltheart et al., 2001), that are processed early and automatically, remain unknown. The scarcity of experimental studies regarding visual word deviance detection may stem from the poor separation of these aspects from lexicality.

If the semantic aspect of a word is processed automatically during lexical processing, response to semantically incorrect word deviation with pre-attentive presentation may manifest as a larger brain activity compared with that manifested by the semantically correct word standard. The Japanese kanji compound, which comprises two or more kanji characters, is useful for investigating this question. The Japanese language is written in kana phonograms, which are analogous to the alphabet, and ideograms called kanji, which are processed by whole-word reading (Dylman and Kikutani, 2017). These ideograms often appear in the form of a kanji compound. A kanji compound comprises a certain combination of kanji, and an incorrect combination is non-lexical as a kanji compound even if the individual kanji characters might be lexical. A kanji compound is thus analogous to a compound word in English—for instance, “sunflower” is lexical, but “sonflour” and “solarflower” are non-lexical. However, kanji compounds commonly represent not just concepts but also their associative formation. Hence, this characteristic of kanji compounds can be used as an experimental condition for semantically erroneous deviation. Importantly, because kanji highlights reading and/or writing disabilities in Japanese, knowledge regarding visual kanji recognition contributes to the understanding of the mechanisms underlying fluent reading ability. Kanji characters have multiple pronunciations and are visually complex, whereas kana characters have one-to-one correspondence to sound and are visually simple (Uno et al., 2008; Higuchi et al., 2020). For these reasons, we focused on the automatic processing of visual kanji compounds. Hence, the semantic processing of lexicality should influence the automatic detection of deviance in kanji compounds.

Although the brain regions associated with automatic lexical processing—particularly pre-attentive visual word deviation detection—are also unknown, the left occipitotemporal regions are expected to play an important role (Woolnough et al., 2020). Neuroimaging studies have reported that visual word recognition is initiated from the primary visual cortex and that the left mid-fusiform gyrus (McCandliss et al., 2003; Dehaene and Cohen, 2011; Lerma-Usabiaga et al., 2018) is robustly activated upon the presentation of a word. One hybrid study using ERPs and functional magnetic resonance imaging demonstrated that the left fusiform gyrus is associated with unconscious visual word recognition and showed an enhanced ERP within 240 ms of stimulus onset (Dehaene et al., 2001). Japanese kanji and Chinese characters are processed in the left fusiform gyrus in the whole-word reading pathway (Sakurai et al., 2006; Liu et al., 2008). The evoked brain response, as evaluated by magnetoencephalography (MEG), for visual word stimuli has been shown to peak in the left inferior occipitotemporal region at 130–150 ms following stimulus onset and to be larger than that for noise images or letter/syllable representations (Tarkiainen et al., 1999). These studies suggest that the left occipitotemporal regions are important for the automatic detection of kanji compound deviation within 300 ms of presentation.

Here we aimed to investigate three questions. First, whether there is an automatic detection of kanji compound deviation. Next, to see whether visual or lexical deviation can be automatically detected. Finally, we analyzed which visual areas are associated with each type of processing. To achieve these aims, we measured the brain activity of the areas related to visual word recognition. We examined automatic deviance detection in three types of sequences: visual feature deviance (identical kanji compounds presented using different fonts), lexical deviance (lexically correct vs. incorrect kanji compounds) and non-word figural deviance (difference among pseudo-kanji character figures). The sequences comprised 20% deviants and 80% standards and were presented in the parafoveal vision while the participants fixated on a silent movie presented in the fovea to divert attention away from the stimuli for measuring automatic detection. We set the differences in the stimuli frequency to emphasize each stimulus included in the sequences.

Thus, we employed an inattentive oddball paradigm to probe automatic deviance detection. This experimental protocol is similar to that for mismatch negativity (MMN) (Sams et al., 1985). In fact, visual MMN (vMMN) is often referred to as equivalent to visual deviance detection, and therefore, we have cited related studies in this paper. However, in the present study, we will not use the term vMMN to describe deviance detection because we are not sure if our process of concern is vMMN at present. MMN is an ERP that is often used for studying the automatic detection of deviance (Turker and Hartwigsen, 2021). It is a negative-going ERP component observed when a mismatch of events between the prediction—based on sequential regularity—and the actual stimulus that may contain unanticipated deviants are presented. It is commonly studied using a paradigm wherein a sequence of frequent standard stimuli with that of infrequent and unexpected deviant stimuli (oddball) is presented inattentively. The deviant stimulus leads to negativity with larger amplitude than the standard stimuli. Previous studies subtracted the standard waveform from the deviant waveform to derive the MMN and vMMN and reported that the vMMN can be elicited by simple sensory features (such as color, line orientation and spatial frequency) as well as at highly cognitive levels (such as simple geometric patterns, facial emotions and lexical processing) (Stefanics et al., 2014). Although the vMMN may result from a variety of unintentional temporal context-based predictions (see {Kimura, 2012 #20; Stefanics et al., 2014 #58} for reviews), the notion of “genuine” vMMN still remains debatable. Auditory MMN is considered to be elicited by deviation from the prediction based on sensory memory trace, which cannot be controlled easily in the visual modality of ERP measurements (Male et al., 2020). Thus, it is unclear whether the prediction based on the temporal context in the sensory memory solely explains the visual response analogously to the auditory modality. There can be the effects of temporal and spatial contexts as well as spatiotemporal context and more cognitive constraints and their interactions. Because we aimed to elucidate the mechanisms underlying reading, including the recognition of the unattended word stimuli in the peripheral visual field, we will not limit our discussion to the vMMN in the current study. The relationship between the response and vMMN will be addressed in subsequent studies.

We hypothesized that preliminary processing in the primary visual cortex prior to word-specific recognition contributes to automatic font-type deviance detection because the font-type difference leads to a quantifiable physical deviance without altering the kanji compound identity. In comparison, the automatic deviance detection of lexically correct vs. incorrect kanji compounds would result in significantly different activities in the left occipitotemporal regions because detecting lexical deviation requires access to long-term lexical memory based on the stored representation of kanji compounds. Regarding the lexical deviance sequence, it is possible that the kind of deviation—that is, correct-in-incorrect vs. incorrect-in-correct—shows different sensitivities to automatic deviance detection due to the word superiority effect (Reicher, 1969), and the deviant word is thus detected more easily than the standard word. Furthermore, we used pseudo-kanji character stimuli mimicking correct and incorrect stimuli to isolate the visual word-specific processing. If the significantly larger brain activity in the left occipitotemporal region occurs only for lexical deviance—that is, for correct vs. incorrect kanji compounds—but not for pseudo-kanji character and font-type deviance, it proves that the semantic aspect of kanji compounds is processed early and automatically. We employed MEG to clarify the spatiotemporal dynamic activation in the automatic visual detection of deviant kanji compounds. We collected and analyzed the MEG data *via* the widespread minimum norm estimate (MNE) analysis. In accordance to previous studies related to visual word recognition (Lerma-Usabiaga et al., 2018; Woolnough et al., 2020), we focused on three regions of interest (ROIs) from the primary visual cortex to the occipitotemporal region in the left hemisphere (McCandliss et al., 2003; Dehaene and Cohen, 2011): the occipital pole, posterior transverse region of the collateral sulcus (pCoS) and fusiform gyrus. We performed statistical analysis to examine the deviant effects on visual processing in each sequence. We also examined the effects of word lexicality type (lexically correct kanji compounds, lexically incorrect kanji compounds as meaningless words and kanji-like figures as non-words) to identify the visual word-specific processing influenced by lexical information in the parafoveal vision, which is thought to provide the foveal vision with information for fluent reading (Schotter et al., 2011).

## Results

### Root mean square analysis for sensor signals

**FIGURE 1:**
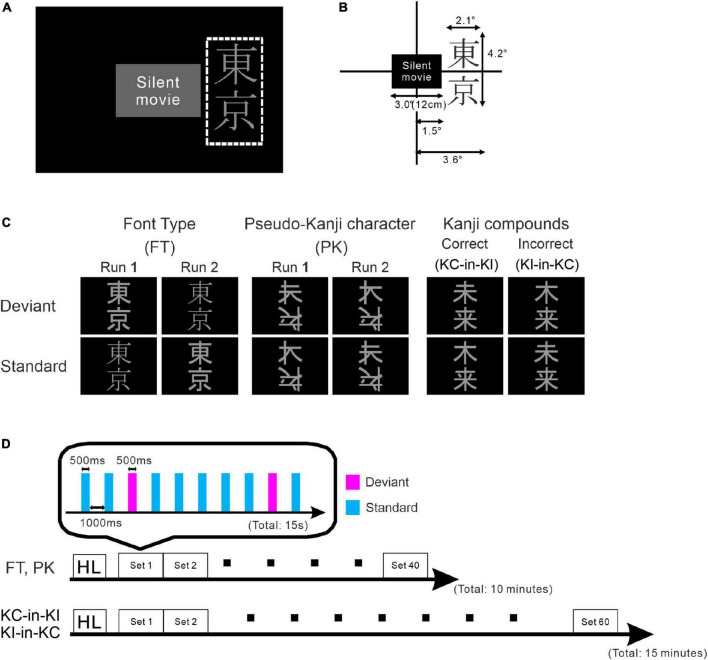
Schematic drawing of the stimulus and paradigm. **(A)** Stimulus layout. The white dotted rectangle indicates the display area for the stimulus oddball sequence. The participants watched silent movies at their central fovea and the oddball sequences were presented in their parafoveal vision. **(B)** Details of stimulus size. **(C)** Stimulus images: font-type change sequences (FT), pseudo-kanji character change sequences (PK), correct-in-incorrect kanji compound sequences (KC-in-KI) and incorrect-in-correct kanji compound sequences (KI-in-KC) from left to right. **(D)** Sequence of each run. One blocked set comprised 10 presentations, 8 standards (cyan) and 2 deviants (magenta). The order of stimulus presentation or standard and deviant trials was randomized. The FT and PK sequences comprised 40 blocked sets per run and the KC-in-KI and KI-in-KC sequences comprised 60 blocked sets per run. The participants took short breaks of approximately 5 min between successive runs.

**FIGURE 2:**
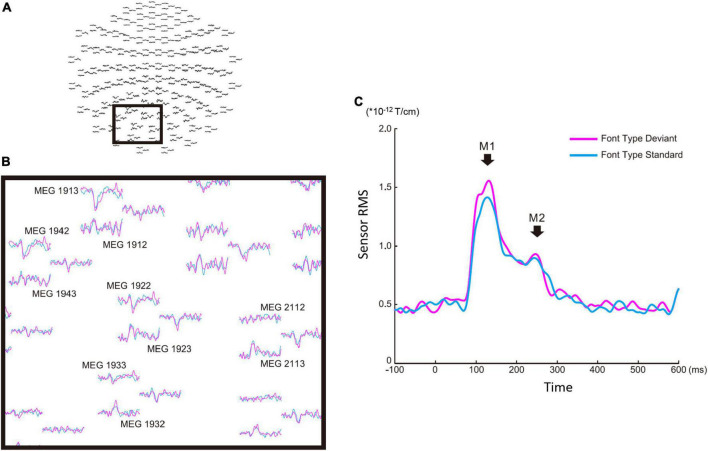
Sensor level analysis of the font-type change condition. **(A)** Sensor maps of all 204 gradiometers of a representative participant. The gray square indicates the sensors of interest. **(B)** Left occipital sensors enlarged from the gray square marked in **(A)**. **(C)** Root mean square waveforms for the deviant (magenta) and standard (cyan) cases. The M1 and M2 peaks were identified from individual participants’ waveforms to define the time window common across the participants for source analysis.

There were four sequences of three types used in this study: the font-type change sequences (FT), correct-in-incorrect kanji compound sequence (KC-in-KI), incorrect-in-correct kanji compound sequence (KI-in-KC) and pseudo-kanji character change sequences (PK). Figure 1 shows these sequences schematically (see “Materials and methods” section for details). After applying filters and a noise elimination process, at least 114 trials remained and were averaged for each sequence. We first performed a sensor level analysis using the averaged signals to identify the main peak responses that defined the time windows. We selected 10 sensors with higher magnitudes for averaging the activities from the left occipital side (Figure 2A). The sensors corresponding to both the dorsal and ventral pathways displayed prominent peaks (Figure 2B). We constructed root mean square (RMS) signals for each sequence by selecting the 10 most responsive gradiometers. Figure 2C (see also Supplementary Figure 1) shows the RMS analysis schematically. Within 300 ms of the stimulus onset, the sensor RMS had two peaks, which we identified as corresponding to the markers in Figure 2C, M1 and M2 peaks. The mean and standard deviation (± SD) of the latency across participants for the M1 and M2 peaks were 124.5 ± 17.0 and 198.9 ± 32.6 ms, respectively. Hereafter, we will focus on these two peaks for source activations. We detected the source activity peaks of each participant within a time window of 80–160 ms for M1 and 160–250 ms for M2.

### Source activity

**FIGURE 3:**
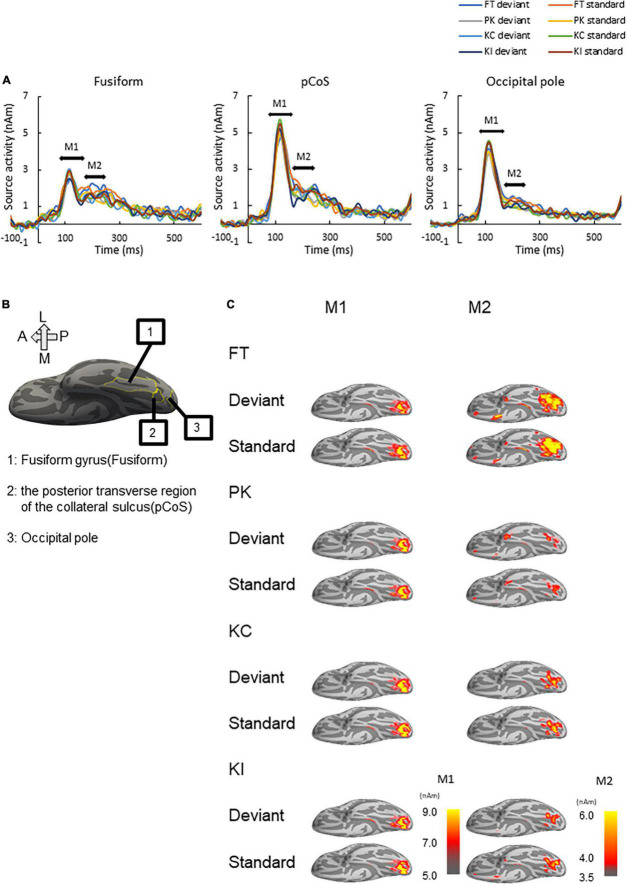
Results of source analysis. **(A)** Source waveforms for the three ROIs [fusiform gyrus, posterior transverse region of the collateral sulcus (pCoS) and occipital pole] for each combination of stimulus [font-type change (FT), pseudo-kanji character figures (PK), correct kanji compound (KC) and incorrect kanji compound (KI)] and frequency (deviant, standard). **(B)** Anatomical position of the three ROIs. **(C)** Peak activation maps of the ventral view of the left hemisphere for each combination of frequency (deviant, standard) and stimulus (FT, KC, KI, and PK) for M1 and M2. Pseudocolor indicates the current density in nAm, as indicated by the color scale. We generated peak activation maps by averaging the individual maps at the individual peak latencies after normalization and transformation to the standard brain.

We performed source analysis using the MNE tools. Figure 3A shows the results of the source analysis for all the three ROIs and four stimulus types. M1 and M2 activities were observed within a duration of 100–300 ms. M1 had a clear peak within approximately 80–170 ms, followed by gentle slopes around 180–300 ms as M2. Figure 3B shows the locations of the three ROIs. Figure 3C shows the ventral view of the activation maps of the left hemisphere for each combination of the four stimulus types and the deviant and standard cases. We have represented font-type change, pseudo-kanji character figures, correct kanji compound and incorrect kanji compound with FT, PK, KC and KI, respectively. Activity was observed to spread from the occipital to the mid-central regions.

### Automatic deviation detection response

**FIGURE 4:**
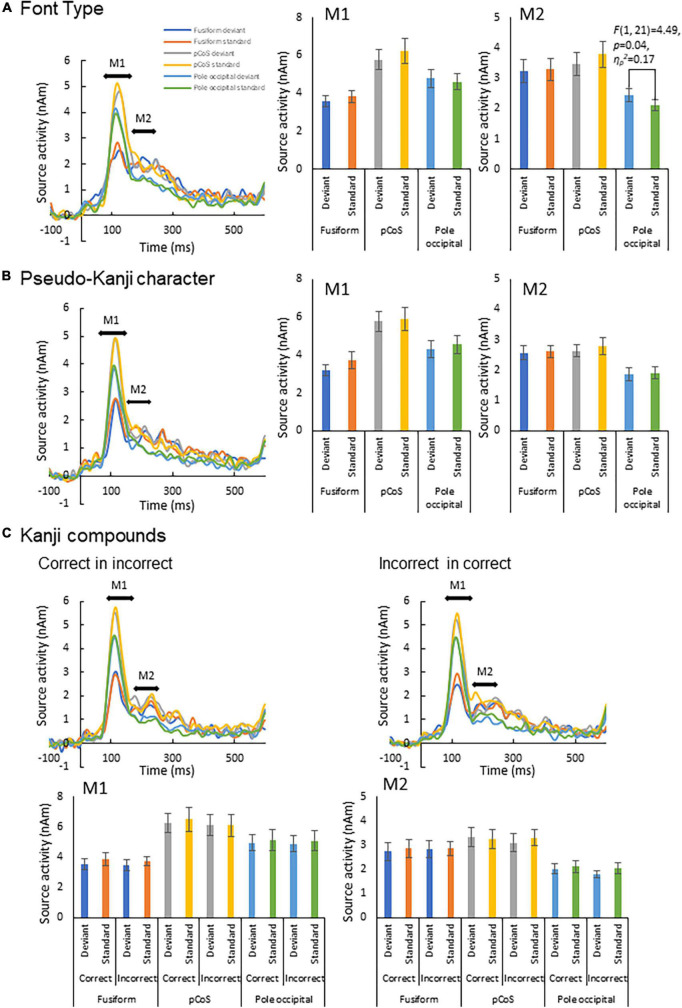
Results of automatic deviation detection response. **(A)** Source waveforms for the font-type change sequence for each combination of the three ROIs (fusiform gyrus, posterior transverse region of collateral sulcus and occipital pole) and frequency (deviant, standard) and the corresponding M1 and M2 peak amplitudes, from left to right. **(B)** Source waveforms for the pseudo-kanji character sequence. **(C)** Source waveforms for the correct-in-incorrect kanji compound and incorrect-in-correct kanji compound sequences.

We subjected the M1 and M2 peak amplitudes for FT and PK to two-way analysis of variance (ANOVA) with three ROIs and frequency (deviant vs. standard cases) as factors. We concatenated the M1 and M2 peak amplitude data between KC-in-KI and KI-in-KC to form a new dataset termed “KC-and-KI” and subjected them to three-way ANOVA with ROI, frequency [deviant vs. standard cases irrespective of correct kanji compound (KC) or incorrect kanji compound (KI)] and lexicality (KC vs. KI irrespective of deviant or standard) as factors. In all the analyses, the effect of ROI was significant. Specifically, the M1 peak amplitude of the pCoS was larger than those of the other two ROIs for all the four stimulus types; the occipital pole showed larger activity than the fusiform gyrus for FT, KC, and KI, but was not significantly different for PK [FT: *F*(2, 42) = 15.3, *p* < 0.001, η*_*p*_*^2^ = 0.42; KC-and-KI: *F*(2, 42) = 12.84, *p* < 0.001, η*_*p*_*^2^ = 0.37; PK: *F*(2, 42) = 13.33, *p* < 0.001, η*_*p*_*^2^ = 0.38]. The M2 peak amplitude of the pCoS was larger than that of the occipital pole for all the four stimulus types, but the fusiform gyrus showed larger activity than the occipital pole for FT, KC-and-KI and PK [FT: *F*(2, 42) = 10.67, *p* = 0.0002, η*_*p*_*^2^ = 0.33; KC-and-KI: *F*(1.5, 33.1) = 14.40, *p* = 0.0001, η*_*p*_*^2^ = 0.40; PK: *F*(2, 42) = 9.68, *p* = 0.0003, η*_*p*_*^2^ = 0.31]. There were no significant differences between M1 or M2 for the other factors—namely, frequency [M1-FT: *F*(1, 21) = 0.99, *p* = 0.32, η*_*p*_*^2^ = 0.04; M1-KC-and-KI: *F*(1, 21) = 1.77, *p* = 0.19, η*_*p*_*^2^ = 0.07; M1-PK: *F*(1, 21) = 1.82, *p* = 0.19, η*_*p*_*^2^ = 0.08; M2-FT: *F*(1, 21) = 0.008, *p* = 0.92, η*_*p*_*^2^ = 0.0004; M2-KC and KI: *F*(1, 21) = 0.55, *p* = 0.46, η*_*p*_*^2^ = 0.02; M2-PK: *F*(1, 21) = 0.56, *p* = 0.46, η*_*p*_*^2^ = 0.02] and lexicality [M1-KC and KI: *F*(1, 21) = 0.91, *p* = 0.35, η*_*p*_*^2^ = 0.04; M2-KC-and-KI: *F*(1, 21) = 0.31, *p* = 0.58, η*_*p*_*^2^ = 0.01]. We observed a significant difference between the deviant and standard cases only in the M2 of FT at the occipital pole area (Figure 4; see also Supplementary Figures 2, 3), which was a significant interaction effect between ROI and stimulus frequency [*F*(2, 42) = 3.98, *p* = 0.02, η*_*p*_*^2^ = 0.15]. *Post-hoc* tests showed that the M2 peak amplitude of the deviant cases was larger than that of the standard cases [*F*(1, 21) = 4.49, *p* = 0.04, η*_*p*_*^2^ = 0.17]. The M2 peak of the deviant case showed larger activity in the pCoS than in the occipital pole, whereas the M2 peak of the standard case showed larger activity in the pCoS and fusiform gyrus than in the occipital pole region for the FT sequences.

### Type-specific response in the posterior transverse region of the collateral sulcus

**FIGURE 5:**
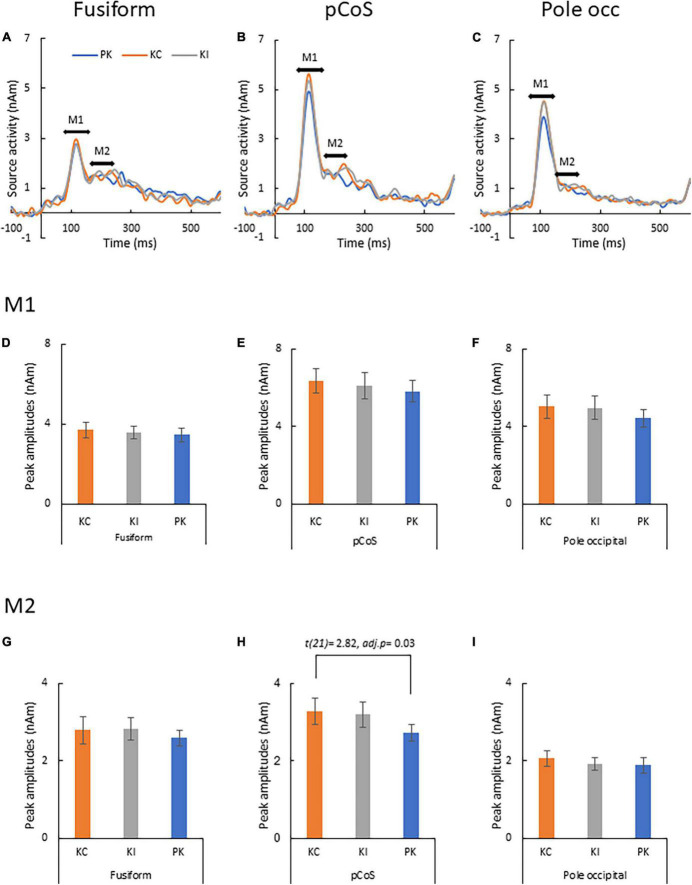
Results of type-specific responses. **(A)** Source waveforms in the fusiform gyrus for the three stimulus types [pseudo-kanji character (PK), correct kanji compound (KC) and incorrect kanji compound (KI)]. **(B)** Source waveform in the posterior transverse region of the collateral sulcus (pCoS). **(C)** Source waveform in the occipital pole. **(D)** Mean M1 peak amplitude in the fusiform gyrus (fusiform) for the three sequences: correct kanji compounds (KC), incorrect kanji compounds (KI) and pseudo-kanji character (pseudo). **(E)** Mean M1 peak amplitude in pCoS for the three sequences. **(F)** Mean M1 peak amplitude in the occipital pole (occipital pole) for the three sequences. **(G)** Mean M2 peak amplitude in the fusiform for the three sequences. **(H)** Mean M2 peak amplitude in pCoS for the three sequences. **(I)** Mean M2 peak amplitude in the occipital pole for the three sequences.

Figure 5 presents the results of the source analysis for making lexical comparisons between the different stimulus types. Figures 5A–C (see also Supplementary Figure 4) shows the comparison of the source waveforms in the three ROIs: the fusiform gyrus, pCoS and occipital pole, respectively. The latencies of the main peaks concentrated at around 115 ms, which suggested M1 peaks for all the three types. The relatively smaller M2 peaks appeared following the M1 peaks. Figures 5D–F present the results of statistical analysis for the M1 mean amplitudes across the participants. There was no statistically significant difference in the M1 peaks after comparison between the stimulus types [occipital pole: *F*(1.1, 24.3) = 1.89, *p* = 0.18, η*_*p*_*^2^ = 0.08; pCoS: *F*(1.4, 30.0) = 0.74, *p* = 0.44, η*_*p*_*^2^ = 0.03; fusiform gyrus: *F*(2, 42) = 0.48, *p* = 0.61, η*_*p*_*^2^ = 0.02]. Figures 5G–I show the results of statistical analysis for the M2 peak amplitudes across the participants. ANOVA indicated a significant main effect of the stimulus type in the pCoS [Figure 5H, *F*(1.5, 32.3) = 5.4, *p* = 0.01, η*_*p*_*^2^ = 0.20] but not in the other ROIs [fusiform gyrus: Figure 5G, *F*(1.6, 33.9) = 0.49, *p* = 0.57, η*_*p*_*^2^ = 0.02; occipital pole: Figure. 5I, *F*(2, 42) = 1.3, *p* = 0.28, η*_*p*_*^2^ = 0.05] after applying Holm’s sequentially rejective Bonferroni procedure for multiple comparisons. *Post-hoc* tests showed that the M2 peak amplitude in the pCoS was significantly larger for the correct stimulus than that for the pseudo-kanji character stimulus [*t*(21) = 2.82, *adj.p* = 0.03, corrected using the Holm-Bonferroni method].

## Discussion

In the present study, we aimed to elucidate the nature of the automatic visual detection of deviant kanji compounds and the associated brain activity profiles. We measured the MEG signals while presenting the stimulus sequences to the participants’ parafoveal vision while foveally presenting a silent movie. The results revealed that font-type deviance led to a larger activity in the visual cortices at the early stages (Figure 4). But the lexical deviation of the kanji compound (correct/incorrect) did not lead to a larger activity in any ROI. The pCoS showed a differential activation between the stimulus types, particularly between the correct kanji compound and the pseudo-kanji characters. Even the fusiform gyrus did not respond differently between the correct and incorrect kanji compounds or between the kanji compounds and pseudo-kanji characters. Below, we discuss our findings from two points of view: automatic deviance detection and word type-specific responses.

First, we detected an enhanced response for deviant stimuli compared with that for standard stimuli only in the occipital pole region for the font-type deviance sequence (Figure 4). This result indicates that a change in the visual feature (that is, stroke thickness) of an identical kanji compound that does not affect the lexical aspects is processed automatically by the primary visual cortex. In the font-type change sequence, the varying thicknesses of the stimulus strokes affected the physical quantity of the stimulus, as reflected by the gray level (Table 1). The automatic detection of deviance in the visual modality has been reported for several low-level visual features (Kimura, 2012). Notably, these activity sources include several occipital regions (Kimura et al., 2010; Urakawa et al., 2010; Kogai et al., 2011; Susac et al., 2013). Our observation is consistent with a previous report regarding the automatic detection of deviance defined by luminance changes in circle stimuli (Kimura et al., 2008). As hypothesized, this response to the font-type difference should reflect the preliminary processing of the parafoveally and pre-attentively presented kanji compound. In contrast, we identified no clear response of deviance detection for lexical and pseudo-kanji characters (Figure 4). There are two potential explanations for this result: Deviation saliency and attention level. Compared with that of previous studies, the deviation saliency of the stimulus sequences used in this study was lower. For example, Wei et al. (2018) reported the detection of automatic lexical processing in Chinese by native readers using Chinese characters vs. pseudo-Chinese characters. Unlike Wei et al. (2018), we contrasted between correct and incorrect kanji compounds in the stimulus sequence used in this study. It may have led to the low deviation saliency. Also, in the current study, the deviance in the pseudo-kanji character pairs was not automatically detected. As shown in Figure 1C and Table 1, the kanji compounds and pseudo-kanji character stimuli were visually complex, but the differences based on the strokes in the stimuli were small and not salient. Previous studies using complex figures as stimuli have reported deviation detection responses among visually simple—and therefore salient—standard stimuli, such as oblique bar patterns (Kojouharova et al., 2019). In regards to attention level, previous automatic deviance detection studies with and without the use of visual word stimuli also required an active sub-task, such as detecting a dummy sub-target and pressing a button in the participant’s foveal visual field, to divert the participant’s attention from the main target (Kimura et al., 2008, 2010; Kogai et al., 2011; Shtyrov et al., 2013; Susac et al., 2013; Wei et al., 2018; Kojouharova et al., 2019). In the present study, the participants passively watched a silent movie. Although such a dummy sub-task reduces attention to the stimulus, it helps maintain the participants’ arousal. A previous study that also used passive watching of a silent movie (Urakawa et al., 2010) showed an automatic detection of color change (red/blue) deviation in the middle occipital gyrus. Compared with detecting color change, detecting deviation in kanji compounds and pseudo-kanji characters may be more difficult because of the stimulus complexity, and hence, may require a higher level of arousal or more general attention to the participant’s wider visual field. Detecting deviation in the kanji compound may require effort-demanding processing and may therefore not be automatic. It may be difficult to form distinctive expectations about deviations because of its complexity.

**TABLE 1:** All stimuli and luminance values (cd/m^2^).

Stimuli sequences	Word types	Luminance values (cd/m^2^)	
Font type	Sans-serif gothic	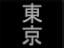	5.30
	Serif mincho	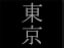	1.98
Kanji compounds	Correct	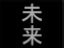	5.97
	Incorrect	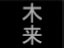	6.06
Pseudo-kanji character	Pseudo-kanji 1	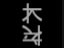	4.03
	Pseudo-kanji 2	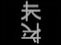	4.22

Second, we observed a word type-specific response in our study. Regardless of the stimulus frequency, the correct kanji compounds showed larger source activities than the pseudo-kanji characters in the pCoS, which is located in a fusiform neighboring region. In contrast, the activity for the incorrect kanji compounds and the other two stimuli—namely, the correct kanji compounds and pseudo-kanji characters—did not differ (Figure 5). This result indicates that although native Japanese speakers can detect learned kanji compounds, they cannot identify the lexical error in the parafovea within 250 ms of stimulus onset. Studies regarding word type-specific response showed specific time windows and brain regions for the different levels of lexicality when the participants paid attention to the foveally presented word or pseudo-word stimuli. Ramin and Pulvermüller showed that word length (physical factor) is processed within 100 ms while the first word frequency-related responses (non-physical factor, genuine word-familiarity) appeared 120–160 ms after stimulus onset (Assadollahi and Pulvermüller, 2001). Hauk et al. (2006) showed in an EEG study that lexical frequency (words vs. unpronounced words) affected the waveform at 110 ms after stimulus onset and that semantic coherence correlated with the activity from the lexical frequency effect at approximately 160 ms. Thesen et al. (2012) reported that letter- and word-selective responses occurred 160 and 225 ms after stimulus onset, respectively, and that the brain source of the word-selective response was at a more anterior location than that of the letter-selective response. Deviations from semantic memory—such as context or word category—are known to be processed in the cognitive processing indicated by N400 (Kutas and Federmeier, 2000), although the detection of a word lexical error without reference to semantic memory did not occur within 250 ms. Similarly, parafoveal word recognition within 300 ms seems to be sensitive to the word lexical frequency but not semantic errors. Studies on eye movements during reading showed that the parafoveal word frequency relates to the processing speed of the foveal target word, with longer gaze duration for the low frequency target word compared with the high frequency target word (Schotter et al., 2011; Li et al., 2015; Rusich et al., 2020). Pan, Frisson and Jensen reported that larger tagging responses occurred during pre-target fixations, followed by low—compared with high—lexical frequency targets (Pan et al., 2021). Dimigen et al. (2012) found that target words with identical pre-target led to shorter gaze duration, which was reflected by a positively directional ERP between 200 and 280 ms at an occipitotemporal site. These studies show that the word’s lexical frequency is processed between 160 and 250 ms, later than the physical or other sub-lexical aspects (for instance, pronunciation) which is processed between 100 and 160 ms, and the lexical frequency processing is facilitated by prior parafoveal presentation. In the present study, significant differences related to word types were not found within 160 ms but 160–250 ms of stimulus onset. This suggests that some form of lexical frequency differences between the three stimuli—KC, KI and PK—resulted in the word type-specific response. The PK—but not the KI—was significantly different from the KC, maybe because only the PK was unpronounced and showed a larger contrast with the KC compared with the infrequent but pronounced KI.

In the current study, we did not observe a word-specific response in the fusiform gyrus, the key brain region associated with visual word recognition; however, we found the fusiform neighboring area, the pCoS, to be involved in the discrimination of words from non-words. Several recent studies have identified brain areas that are posteriorly adjacent to the left mid-fusiform gyrus and appear to perform letter-specific processing rather than word-specific processing (Thesen et al., 2012; Gwilliams et al., 2016; Lerma-Usabiaga et al., 2018; Lochy et al., 2018; Woolnough et al., 2020). Thesen et al. (2012) reported that letter-selective responses began at 160 ms after word onset, which is earlier than word-selective responses. This finding suggests that words are first encoded as letters during reading before being encoded as word chunks. It is possible that our observation regarding the pCoS activity specific to the lexically correct kanji compounds relates to the letter-specific area or activation in the early visual areas for the preliminary processing of letters and words (Tarkiainen et al., 1999; Caspers et al., 2014; Gagl et al., 2020; Heilbron et al., 2020; Woolnough et al., 2020). Alternatively, it may relate to differential recognition between learned words and non-word non-letters—that is, irrelevant stimuli in the parafovea. The foveal letter-specific activity and alleged parafoveal word-specific activity in the fusiform neighboring region may together underpin foveal word recognition in the fusiform gyrus. Considering that contextual predictions affect the activity of the early visual cortex, including the primary visual cortex (Heilbron et al., 2020), our results imply that the orthographic information of learned compounds is in fact processed automatically. Although compound processing may not result in the detection of lexically incorrect deviants, it could lead to the pre-attentive detection of deviance in the low-level visual features (i.e., font differences) and discrimination between lexically correct compounds from non-linguistic scripts (i.e., pseudo-kanji characters). Children who are still learning to read and people with reading disabilities may not be able to automatically process any orthographic information due to incomplete orthographic information storage.

The present analysis based on differential peak amplitudes using source estimates might have led to different results compared with the results of several previous studies that utilized mismatch negativity based on subtraction between the standard and deviant waveforms. However, we calculated the differential peak amplitudes on individual waveforms but not on the averaged waveform because the averaged waveform did not show a clear peak response. It is thus likely that the averaged subtraction waveform did not show a prominent negativity that could be reliably considered mismatch negativity. We cannot analyze individual subtraction waveforms because there are no valid statistics to evaluate regarding whether the individually identified negative maxima are non-zero.

In the present study, we selected phase-locked millisecond-range responses as the indices of deviation detection. Conversely, non-phase-locked responses of at least 20 ms duration and up to 1,000 ms, such as high-gamma augmentation, are also used as an index of the reading-related responses in intracranial recordings. For example, Wu et al. (2011) reported that word reading elicited gamma augmentation (maximally at 80–100 Hz) using electrocorticography in the occipital, temporal, parietal and Rolandic areas. Gamma augmentation at 50–120 Hz in the bilateral occipital and inferior/medial–temporal regions reflected naming to visual stimuli (Kojima et al., 2013). These studies suggest that reading-related cognitive activities have two aspects, transient and sustained. It is possible that an index reflecting more long-lasting stable activities than those of the current index—such as high-gamma augmentation—could reveal a clearer difference in an aspect of the automatic visual detection of deviant kanji compounds.

Recent studies have reported the successful application of brain stimulation to reading disabilities (Rufener et al., 2019; Hanslmayr et al., 2020). Stimulation was targeted at the auditory cortex in these studies, potentially based on the grapheme–phoneme association as the basis for reading acquisition in alphabetical languages; however, characters may not be associated with sound alone in a different language. Chinese characters that are also used in Japanese writing as kanji represent meaning in addition to pronunciation. This difference indeed seems reflected by the developmental shift of the areas activated by reading from the auditory network in comparison to the visual network (Zhou et al., 2021). The stimulation may be more effective when it is provided to the areas outside of the auditory association cortices for those who write using Chinese characters. This issue is most complicated with Japanese because there are two very different sets of characters—the kana phonograms for sound and the kanji ideograms for meaning—in its writing system. The neural representation of the Japanese writing system may need further investigation to develop applications for the brain stimulation of patients with difficulties in reading in Japanese.

This study is subject to several limitations. First, there was no variability in the compound word stimulus for each type of sequence to ensure sufficient averaging for the deviants in a short time. Because semantic deviation between the correct and incorrect kanji compounds was not salient and there was no task demand for the participants’ attention, maintaining the participants’ arousal level would have been difficult if there were many repetitions of the sequences. Although the present paradigm was not ideal, we optimized it for the main purpose of this study—to elucidate the aspects of visual word recognition that can be automatically processed, bearing pediatric applications in mind. Thus, we prioritized the variety of deviations. Second, the sub-task (that is, the movie) had to be free from transient on-offs to measure the occipital pole activity in response to the main task (namely, the sequences). As previous studies have found the participants’ general level of attention to the visual field to affect visual word recognition, the effects of attention levels should be investigated in future studies. Third, this study examined event-related processing in a limited frequency band, therefore did not exhaustively explore all possible signal regions or pre-processing techniques that could be included in the analysis: e.g., responses with long latencies after 250 ms or in different frequency bands. This is an issue for future studies. Lastly, the observed font-type difference effects should also be tested using lexically incorrect or pseudo-kanji characters to determine the effects of long-term lexical memory on the automatic detection of font-type differences. In the present study, because we assumed that the detection of font differences was a lower-order process than semantic processing, we did not question the ability to detect such deviations irrespective of lexicality. If higher-order processes are more involved than we expected, participants with a reading disability may not be able to detect font deviations.

To conclude, we found the automatic detection of deviant kanji compounds when they were changed visually, but not semantically or orthographically. The detection of the visual change of kanji compounds stimulated activity in the occipital pole area. In addition, even being presented in parafovea without direct attention, kanji compounds could be distinguished from the pseudo-kanji character figures in an area which neighbors the fusiform gyrus. Our results suggest that the automatic visual recognition of kanji compounds is limited to a low-level feature in people without reading disabilities.

## Materials and methods

### Participants

We recruited 22 healthy undergraduate and graduate students who were all native Japanese speakers (age 20–26 years, 6 males and 16 females) for this study. All the participants were right-handed according to the Edinburgh handedness inventory (Oldfield, 1971) and showed normal or corrected-to-normal visual acuity. We excluded individuals who were under treatment for or having been diagnosed with psychiatric disorders during the recruitment. We confirmed the intact reading and writing skills of all the participants using the Japanese version of the Kaufman Assessment battery for children-second edition (K-ABCII; Kaufman and Kaufman, 2013) subtests (reading and writing) conventionally, although the examination should be used on children aged 18 years or younger.

### Stimuli and procedure

The stimulus sequence was generated using SuperLab 5 software (Cedrus Corporation, San Pedro, CA, United States) and presented through a hole in the wall onto a back-projection screen placed 226 cm in front of the participant inside a magnetically shielded room. A DLP projector (TAXAN KG-PS233X, Kaga Electronics, Tokyo, Japan) with a resolution of 1,024 × 768 pixels at 50 Hz was used for stimulus presentation. The participants watched silent movies in their central fovea in parallel with oddball sequences being presented in the parafovea (Figure 1A) without paying direct attention to the stimulus sequence. All stimuli sequences comprised light gray characters displayed on a black background, with visual angles of 2.1° horizontally and 4.2° vertically (Figure 1B) and consisted of two kinds of kanji compounds oriented vertically. Figure 1C illustrates all the sequences and stimuli. We used one pair of stimuli for each condition. Table 1 lists all the six images. In FT, the deviation was generated by a font-type difference and the stimuli were generated from the identical kanji compounds using different font types (Sans-serif Gothic vs. Serif Mincho). We created incorrect compounds, which were included in KI-in-KC as the deviant and in KC-in-KI as the standard, by substituting one of the kanji in the correct kanji compound with a different kanji missing a stroke; they were therefore similar in shape but completely different and unrelated in meaning. This incorrect stimulus reflected a typical pattern of writing mistake in Japanese, which is frequently and specifically made by patients with learning disorders. The kanji compounds used were composed of those learned by the age of 10 years, and hence, should have been familiar to the Japanese-speaking participants who had no difficulty in reading and writing. In PK, the pseudo-kanji characters were created by shifting strokes from the kanji in the correct and incorrect kanji compounds and combined to form the stimulus. The resolution of the kanji compound or the pseudo-kanji character figure was 300 × 240 pixels and the mean gray values of the pixels were as listed in Table 1. Figure 1D illustrates the details of the individual sequence that comprised a run. Each run included 20% deviants and 80% standards. Each stimulus in the run was displayed for 500 ms. The interstimulus interval in a sequence was 1,000 ms (Figure 1D) and the sequence presentation order was randomized. FT and PK included 800 presentations or trials and consisted of two runs, each comprising 400 trials, by exchanging the standard and deviant stimuli to counteract the physical properties of the individual sequences. Each run lasted 10 min. In KC-in-KI and KI-in-KC, there were 600 trials in each run, each of which lasted 15 min. To determine the source activities, we adjusted the number of averaged trials to be the same (see the “Signal processing” section for further details). The participants took a break every 10–15 min. They were required to verbally explain the story of the silent movie that they had watched after the stimulus presentation.

### Statistical analysis of magnetoencephalography source activation data

To elucidate whether the automatic deviance detection of kanji compounds occurs, we performed two-way ANOVA with repeated measurements for the FT and PK sequences. The factors were ROIs (pole occipital, pCoS and fusiform gyrus) and frequency (standard, deviant). For the KC-in-KI and KI-in-KC sequences, to confirm the lexicality effect, we performed three-way repeated measures ANOVA with the factors ROIs, stimulus frequency and lexicality (KC, KI). If we found a significant main effect or interaction, we performed *post-hoc* testing. As an additional analysis, we performed one-way repeated measures ANOVA for the three ROIs with the factor of word lexicality type (KC, KI, and PK). If the main effect was significant, we applied a paired *t*-test with Holm’s sequentially rejective Bonferroni procedure for multiple comparison correction for *post hoc* analysis. In all ANOVAs, we used Mendoza’s Multisample Sphericity Test to test for sphericity and Greenhouse-Geisser’s Epsilon to correct for degrees of freedom when the assumption did not hold. For statistical calculation, we used the Anovakun version 4.8.5 (Iseki, 2020) with R version 4.0.1 (R Core Team, 2018) and Rstudio Version 1.2.5019.

### Magnetic resonance imaging scanning

We performed MRI scans using a 3-Tesla MRI scanner (Siemens 3T Verio, Siemens, Erlangen, Germany) with 32-channel head coils (Siemens, Erlangen, Germany) at NCNP Hospital. High-resolution T1-weighted anatomical images were acquired using a magnetization-prepared rapid acquisition with gradient echo sequence. Imaging parameters were as follows: repetition time (TR) = 1,900 ms, echo time (TE) = 2.52 ms, flip angle = 9 degrees, field of view = 256 × 256 mm, matrix size = 256 × 256, voxel size = 1 × 1 × 1 mm^3^.

### Magnetoencephalography data acquisition

We acquired MEG data inside a magnetically shielded room using a whole-head system comprising 306 sensors arranged in 102 triplets of two orthogonal planar gradiometers and one magnetometer (TRIUX, Elekta Neuromag, Helsinki, Finland) in sitting position. We digitized the locations of three fiduciary points (nasion, left and right auricular points) defining the head frame coordinate system, a set of head surface points and the locations of the four head position indicator coils using an Isotrak three-dimensional digitizer (Polhemus™, Colchester, VT, United States). During acquisition, we bandpass filtered the signals between 0.1 and 330 Hz and the sampling rate was 1,000 Hz.

### Signal processing

For noise suppression and motion correction, we spatially filtered the data using the signal space separation method (Taulu et al., 2003, 2004) with the Elekta Neuromag Maxfilter software to suppress noise generated by the sources outside of the brain. We applied a bandpass filter of 1–40 Hz. Because we were concerned mainly with pre-attentive automatic processing but not attentive and effortful processes—which are better represented by slower components such as P300—we used a relatively high frequency of 1 Hz as the cut-off for the better removal of fluctuating artifacts, as used in several previous studies (Mitsudo et al., 2014; Hironaga et al., 2017; Johnston et al., 2017). To eliminate outstanding artifacts, we excluded trials during the averaging process if they exceeded a rejection threshold of 5,000 fT/cm (8,000 fT) for gradiometer (magnetometer) channels. We controlled the number of averaged trials to 114–120 for both deviant and standard stimuli in both experiments to remove the potential effects of the signal to noise ratio due to the different numbers of trials when calculating the source activities [details as follows (mean ± SD): deviant FT: 119.0 ± 0.2, KC: 120.0 ± 0.0, KI: 120.0 ± 0.0, PK: 120.0 ± 0.0, standard FT:120.0 ± 0.0, KC: 120.0 ± 0.0, KI:119.7 ± 1.2, PK: 119.9 ± 0.2]. For FT and PK, the data from the two runs were pooled before averaging, as described above for each of the standards and deviants, to cancel out the possible effects of specific font or individual pseudo-kanji character.

### Source reconstruction

We co-registered a reconstructed MRI contour with the MEG head coordinate system using head-shape points (Hironaga et al., 2014). We performed source localization for the averaged data using MNE. Specific details of the MNE algorithms are provided elsewhere (Hashizume and Hironaga, 2016). Briefly, we calculated the lead field that models the signal pattern generated by a unit dipole at each location on the cortical surface using a boundary element method. The general MNE solution is given by:


(1) $$W=RL^{T}{\left(LRL^{T}+\lambda^{2}C\right)}^{-1}$$

where *R* is a source covariance matrix, *L* is a lead field matrix, *C* is a noise (sensor) covariance matrix, λ^2^ is a regularization parameter, and *T* denotes the transposed operator. A covariance matrix *C* was created from the -100 to 0 ms pre-stimulus period, a time range also used for the baseline correction of source waveform signals (that is, regional activities). To compensate for inter-individual differences, we used a standardized brain (MNI-305, fsaverage; Montreal Neurological Institute).

### Group analyses

We performed group analysis using MNE according to the methodology used in previous works (Mitsudo et al., 2014; Hayamizu et al., 2016; Hironaga et al., 2017). We targeted three ROIs in the left hemisphere in the h.aparc.a2009s.annot package (Destrieux et al., 2010) included in the FreeSurfer software (Fischl, 2012). We extracted the source waveforms after applying the baseline correction of -100 to 0 ms. We generally compared two signal patterns, one of which was the differentiation of evoked responses caused by the stimulus patterns. We first marked the peaks in each ROI manually to investigate the transient and shift of latencies from ROI to ROI in the visual system *via* visual inspection.

## Data availability statement

The datasets generated and/or analyzed during the current study are available from the corresponding author on reasonable request.

## Ethics statement

The studies involving human participants were reviewed and approved by the Ethics Committee of the National Center of Neurology and Psychiatry. The participants provided their written informed consent to participate in this study.

## Author contributions

YE, YKa, and AG conceived and designed the work. YE and YKa acquired the data. YE and NH analyzed the data. YE, YKa, AG, and MK interpreted the data. YE, HTake, NH, and AG drafted the manuscript. YKa, YKi, MK, NH, HTake, SH, YKan, HTaka, TH, TO, and MI substantively revised the manuscript. All authors read and approved the final manuscript.

## Abbreviations

ANOVA: analysis of variance
ERP: event-related potential
FT: font type
KC: correct kanji compound
KI: incorrect kanji compound
KC-in-KI: correct-in-incorrect kanji compound sequence
KI-in-KC: incorrect-in-correct kanji compound sequence
MEG: magnetoencephalography
MMN: mismatch negativity
MNE: minimum norm estimate
MRI: magnetic resonance imaging
NCNP: National Center of Neurology and Psychiatry
pCoS: posterior transverse region of the collateral sulcus
PK: pseudo-kanji character
RMS: root mean square
ROI: region of interest
TE: echo time
TR: repetition time.
