# Supplementary material for: Detection of deviance in Japanese kanji compound words
Source: Front Hum Neurosci. 2022 Aug 15;16:913945. doi: 10.3389/fnhum.2022.913945 (PMC9421146; doi:10.3389/fnhum.2022.913945)
Supplement: Supplementary file 1 [file Data_Sheet_1.pdf]

Figure S1

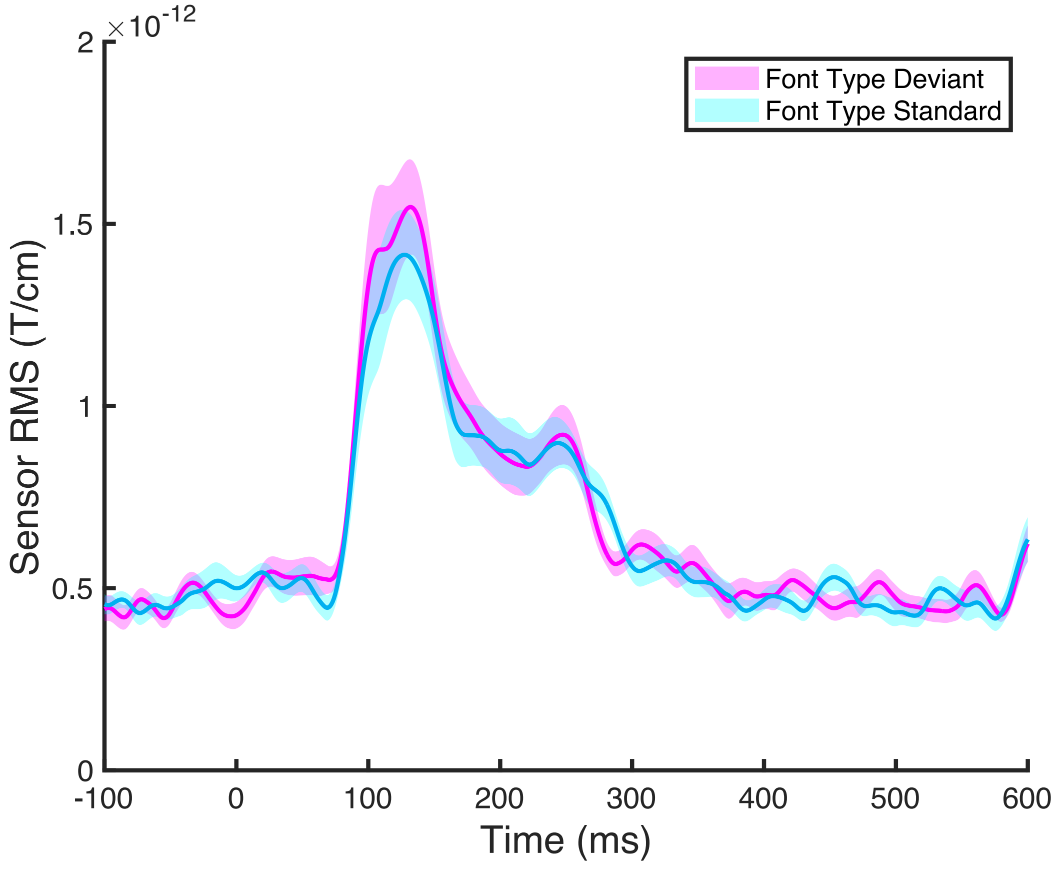

Figure S1. Figure 2C is redrawn with error patches. Results of the sensor level analysis. Root mean square waveforms for the deviant (magenta) and standard (cyan) cases averaged across the 22 participants. The solid line is the mean and the translucent band is the standard error.

Figure S2

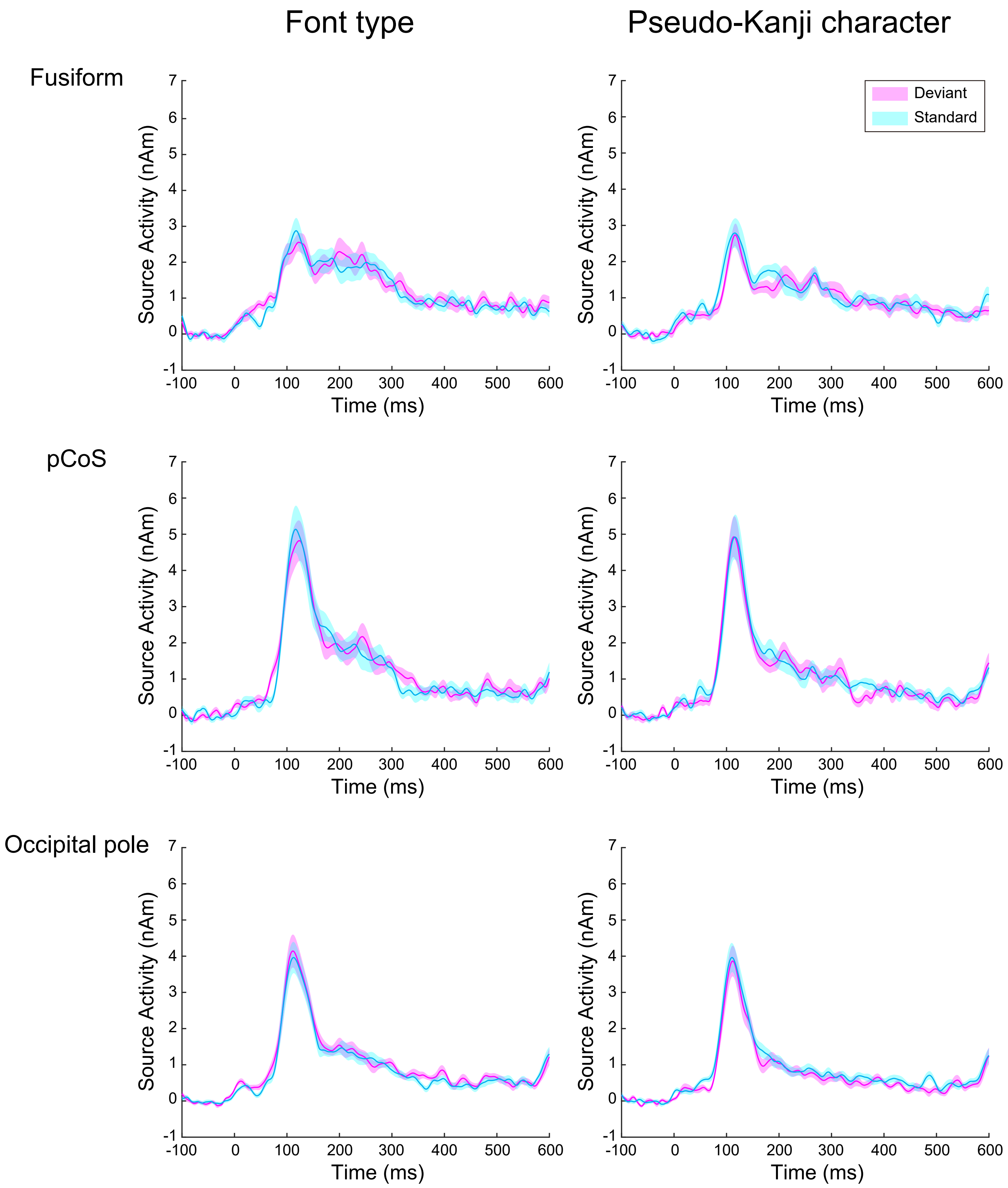

Figure S2. Three ROIs source waveforms for the deviant and standard forms of two word types (font type and pseudo-kanji character). The solid line is the mean and the translucent band is the standard error.

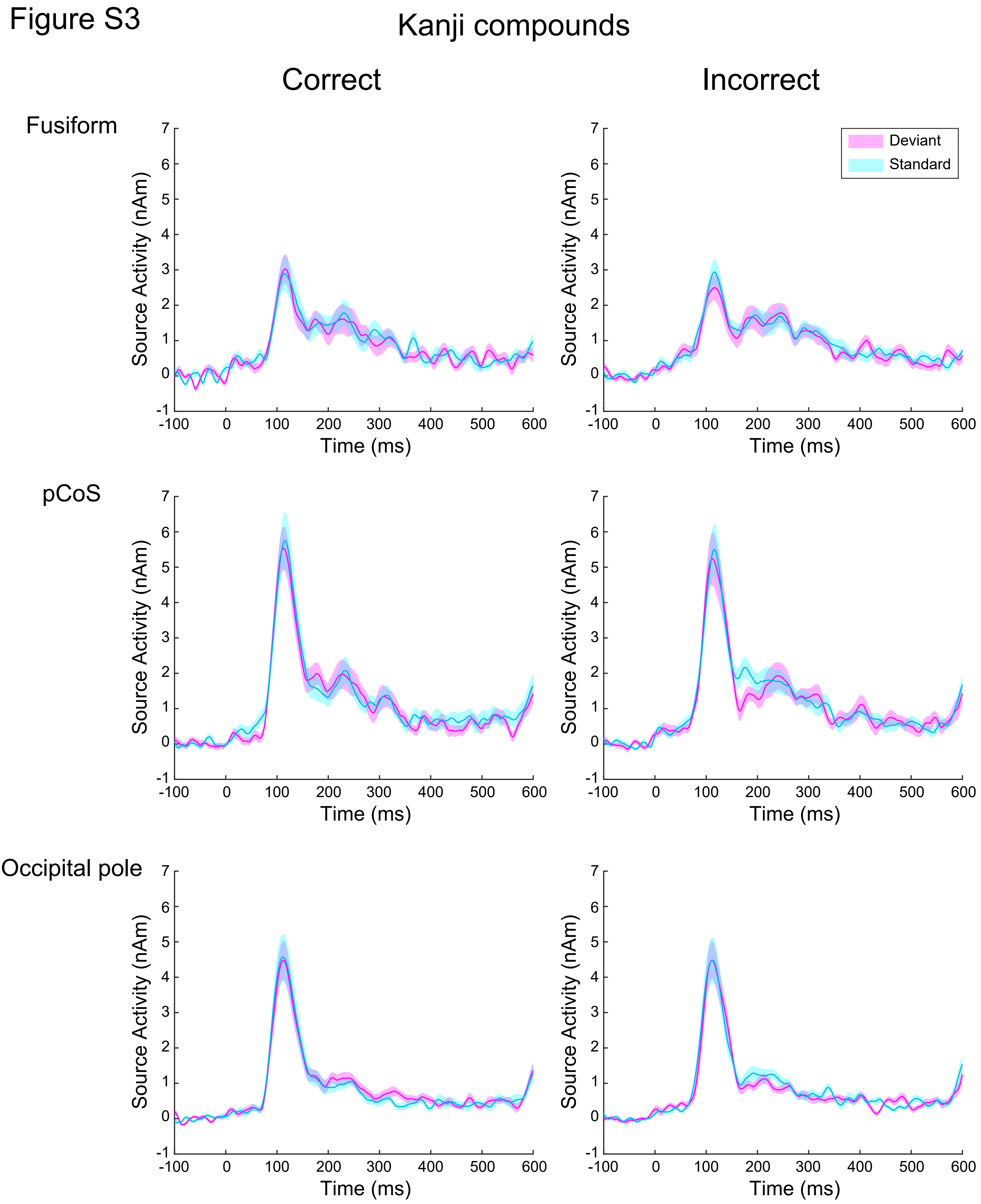

Figure S3. Three ROIs source waveforms of the standard and deviant forms of kanji compounds (correct and incorrect). The solid line is the mean and the translucent band is the standard error.

Figure S4

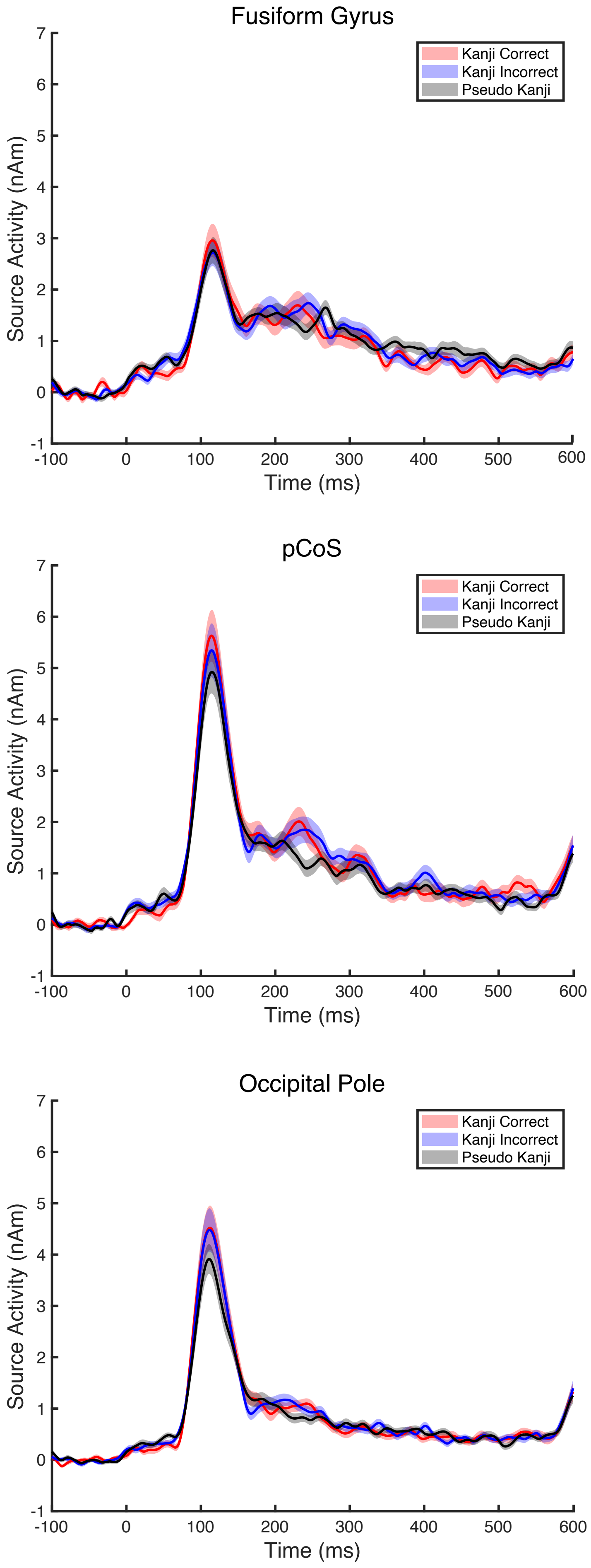

Figure S4. Figure 5 is redrawn with error patches. The patches represent the standard error of mean. Results of the source analysis. Source waveforms for the three ROIs (fusiform gyrus, posterior transverse region of the collateral sulcus (pCoS) and occipital pole) are plotted for each of the three types of stimuli: correct kanji compound (KC), incorrect kanji compound (KI) and pseudo-kanji character figures (PK) after averaging between the standard and deviant cases. The solid line is the mean and the translucent band is the standard error.
